# Interstitielle Keratitis im Rahmen einer Symptomtrias

**DOI:** 10.1007/s00347-020-01241-z

**Published:** 2020-10-16

**Authors:** K. Borgardts, J. Menzel-Severing, M. Roth, R. Guthoff, G. Geerling, K. Spaniol

**Affiliations:** Universitätsaugenklinik Düsseldorf, Moorenstr. 5, 40225 Düsseldorf, Deutschland

## Teil 1: Falldarstellung mit Anamnese, klinischem Befund und Diagnostik

### Anamnese

**Figure 1:**
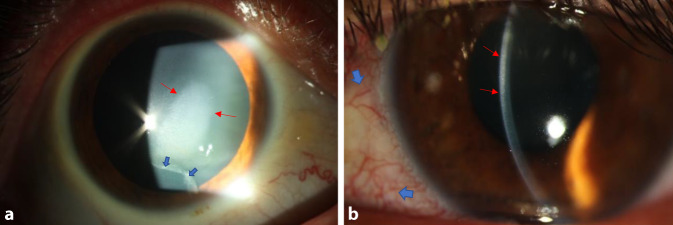

Ein 42-jähriger Mann stellte sich aufgrund rezidivierender Episoden von stechenden und drückenden Schmerzen sowie Fremdkörpergefühl mit Visusminderung auf dem rechten Auge seit etwa 4 Monaten vor (Abb. 1a).

Kurz zuvor habe der Patient an einer Diarrhö unklarer Genese gelitten mit anschließenden Ohrenschmerzen und Hyperakusis auf dem linken Ohr. Einen Tag später sei das rechte Auge schmerzhaft gerötet gewesen. Am Folgetag seien Gleichgewichtsstörungen hinzugekommen, woraufhin er stationär in einer Hals-Nasen-Ohren-Klinik behandelt wurde. Es wurde die Diagnose „Neuritis vestibularis“ gestellt und bei zunehmender Verschlechterung eine systemische Steroidtherapie eingeleitet.

Die bisherige Therapie bestand aus Prednisolonacetat 1 % Augentropfen (AT) seit 2 Monaten bei Bedarf und antibiotikahaltigen AT, die nicht näher benannt werden konnten. Nach Absetzten der steroidhaltigen AT war es stets zu einem Rezidiv der Beschwerdesymptomatik gekommen. Unter der oralen Steroidtherapie, die zum Zeitpunkt der Vorstellung bei 250 mg pro Tag lag, beschrieb der Patient eine Hörverbesserung, weniger Gleichgewichtsstörungen sowie weniger Episoden der okulären Beschwerdesymptomatik. Seit einem Tag seien die Schmerzen und Visusminderung rechts trotz Applikation der steroidhaltigen AT wieder zunehmend. Vier Tage später zeigte sich am Partnerauge die gleiche Symptomatik.

### Befund bei Erstvorstellung

In der klinisch-ophthalmologischen Untersuchung zeigte sich bei einem beidseits bestkorrigierten Visus von 0,8 dezimal am rechten Auge ein zentral gelegenes stromales Infiltrat von ca. 5,5 × 3 mm. Das über dem Infiltrat gelegene Epithel war locker, jedoch geschlossen. Inferior des Infiltrats, im Bereich der sonst klaren Hornhaut, bestand ein Epitheldefekt (Abb. 1a). Es waren keine Endothelpräzipitate zu sehen. Die Vorderkammer war reizfrei und die Linse altersentsprechend klar. Fundoskopisch zeigten sich keine Auffälligkeiten. Der Augendruck lag bei 9 mm Hg rechts, 11 mm Hg links. Am linken Auge, das bei Erstvorstellung reizfrei war, zeigten sich 4 Tage später auch eine diffuse stromale Trübung von 9 bis 12 Uhr mittelperipher und bis an das Zentrum heranreichend sowie eine episklerale Injektion und weißliche intrastromale Immunpräzipitate im anterioren Stroma limbusnah (Abb. 1b).

### Weitere Diagnostik

Eine rheumatologische Mitbeurteilung ergab keinen Hinweis auf eine rheumatische Grunderkrankung. Serologisch ergab sich der Hinweis auf frühere Infektionen mit Herpes-simplex-Viren (HSV) sowie Cytomegalovirus und Immunität gegen Varizella-Zoster. Es zeigte sich kein Anhalt auf eine aktive HSV-Infektion oder Infektion mit dem humanen Immundefizienz-Virus (HIV). Die *Treponema*-Serologie und Borrelien-Antikörper waren negativ. Eine Erhöhung systemischer Entzündungsparameter lag nicht vor. Seitens der Hals-Nasen-Ohren-Ärzte wurde eine hochgradige Innenohrschwerhörigkeit audiographisch gesichert.

## Wie lautet Ihre Diagnose?

### Therapie.

**Figure 2:**
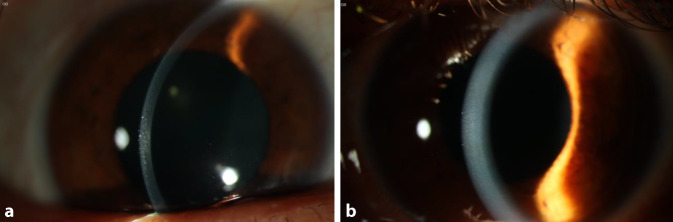

Unter der Therapie mit Ofloxacin 3 mg/ml AT 4‑mal täglich, konservierungsmittelfreiem Tränenersatzmittel mit 0,18 % Hyaluronsäure 5‑mal täglich und Prednisolonacetat 1 % AT 5‑mal täglich zeigten sich der Epitheldefekt und der Reizzustand auf beiden Augen rückläufig. Da bereits über 2 Monate eine topische Steroidtherapie erfolgt war und es nach Absetzen anamnestisch stets zu Rezidiven gekommen war, wurde 9 Tage nach der Erstvorstellung eine Therapie mit Ciclosporin AT 1 % 2‑mal täglich begonnen. Bei Verlaufskontrolle nach 4 Monaten war der Patient unter dieser Therapie symptom- und beschwerdefrei (Abb. 2a, b). Die Hornhäute beider Augen waren bis auf eine hauchige interstitielle Trübung klar bei reizarmen Konjunktiven und reizfreien Vorderkammern (Abb. 2a, b).

## Diskussion

Das Cogan-Syndrom (oder auch okulovestibuloauditorisches Syndrom/Cogan-I-Syndrom) ist eine seltene Erkrankung, die durch eine okuläre nichtinfektiöse Entzündung mit Innenohrschwerhörigkeit und vestibulären Einschränkungen gekennzeichnet ist [1]. Das Cogan-I-Syndrom sollte nicht verwechselt werden mit der okulomotorischen Apraxie (oder auch Cogan-II-Syndrom). Bei der kongenitalen okulären motorischen Apraxie (COMA) kann keine Fixation aufgenommen werden, und das visuelle Verfolgen besonders horizontaler Bewegungen ist nicht möglich, was mit Schleuderbewegungen des Kopfes einhergeht [2].

Im Rahmen des okulovestibuloauditorischen Syndroms sind meist junge Kaukasier betroffen, mit einem Durchschnittsalter von etwa 25 Jahren [3–6]. Espinoza und Prost (2015) berichten von etwa 300 beschriebenen Fällen in der Literatur.

### Krankheitsbild/Symptome.

Die Symptome einer akuten Entzündung sind unspezifisch und können aus Kopfschmerzen, Fieber sowie Gelenk- und Muskelschmerzen bestehen [3, 5]. Bei der typischen Form zeigt sich ein akuter Beginn audiovestibulärer Symptome ähnlich wie bei einem Morbus Menière [3, 4, 6]. Die akustischen und vestibulären Symptome stellen sich klassischerweise beidseits und als plötzlich beginnender Tinnitus, Schwindelgefühl, Ataxie und Übelkeit zusätzlich zu einem Hörverlust dar [3, 4]. Multiple Organsysteme wie die Haut, Nieren, das zentrale Nervensystem und Muskeln können im Rahmen einer mit dem Cogan-Syndrom assoziierten Vaskulitis betroffen sein [3, 5, 6]. Zusätzlich sind eine pleuropulmonale Beteiligung und Lymphadenopathie beschrieben [5]. Histopathologische Untersuchungen der Hornhaut und der Cochlea zeigten eine lymphozytäre und Plasmazellinfiltration, was auf eine zellvermittelte Reaktion hinweist [6].

### Okuläres Bild

Das okuläre Bild bei Cogan-Syndrom ist typischerweise eine interstitielle Keratitis. Die Symptomatik besteht aus reduziertem Visus, Bindehautinjektion, Schmerzen, Fremdkörpergefühl, Epiphora und Photophobie [1, 3]. In der Regel beginnt die interstitielle Keratitis mit subepithelialen Infiltraten [1]. Meist sind beide Augen betroffen bei sehr variabler Symptomausprägung [5]. Das interstitielle Infiltrat zeigt oft ein irreguläres, granuläres Bild und ist bevorzugt im limbusnahen, posterioren Stroma lokalisiert [5]. Bei der atypischen Form können auch eine Panuveitis, (Epi‑)Skleritis oder retinale Vaskulitis vorliegen [1, 3]. Subepitheliale Narben oder Hornhauterosionen können nach der Keratitis beobachtet werden [1, 6], zeigten sich in diesem Fall jedoch simultan.

#### Ätiologie.

Die genaue Pathogenese und Ätiologie des Cogan-Syndroms ist noch nicht vollständig geklärt [3]. Viele Studien bekräftigen eine autoimmune Pathogenese [1, 3, 6]. Betroffene Patienten können Antikörper aufweisen, die eine Ähnlichkeit zu Autoantigenen zeigen wie die im Innenohr und auf Endothelzellen exprimierten SSA/Ro und CD148 [5, 6].

#### Diagnostik.

In Ergänzung zur Spaltlampenuntersuchung können eine Labordiagnostik und Screeninguntersuchungen sinnvoll sein. Oft bestehen eine Leukozytose, Neutrophilie, Thrombozytose, Anämie und erhöhte Blutsenkungsgeschwindigkeit [5, 6]. Zu den Screeninguntersuchungen zählen bei Verdacht auf eine systemische Vaskulitis – die dem Cogan-Syndrom möglicherweise zugrunde liegt – eine Echokardiographie, eine Doppler-Untersuchung sowie eine Angiographie [6]. Zusätzlich sollten ein Audiogramm und ein kalorischer Test u. a. zum Ausschluss eines Morbus Menière durchgeführt werden [4, 6]. Es gibt keinen spezifischen Labortest, der ein Cogan-Syndrom bestätigen oder ausschließen kann [6]. Das Cogan-Syndrom ist daher eine Ausschlussdiagnose. Als mögliche Differenzialdiagnosen sollte unter anderem eine interstitielle Keratitis bei Granulomatose mit Polyangiitis, Polyarteriitis nodosa und rheumatoider Arthritis in Betracht gezogen werden [1].

**Diagnose: **Interstitielle Keratitis bei Cogan-Syndrom

Zusätzlich kann auch eine angeborene Syphilis mit einem Hörverlust und einer interstitiellen Keratitis einhergehen [7]. Eine interstitielle Keratitis im Rahmen der angeborenen Syphilis tritt schleichend auf, zeigt ein limbales Verteilungsmuster und führt häufig zu Hornhautvernarbungen [7]. Schwindel, Übelkeit und Erbrechen werden im Gegensatz zum Cogan-Syndrom nur selten beobachtet [7]. Wichtige Unterscheidungsmerkmale stellen sowohl die positiven serologischen Testergebnisse für Syphilis als auch die systemischen Beteiligungen wie Skelett- und Zahnveränderungen dar [7].

Als weitere mögliche Differenzialdiagnosen infektiöser Ursache sind unter anderem eine Infektion mit Chlamydien, Tuberkulose, Herpes-simplex-Virus, Varizella-Zoster-Virus sowie Borrelien auszuschließen [1, 4, 7, 8].

#### Therapie.

Bislang gibt es keine leitlinienartige einheitliche Therapieempfehlung [3]. Die Behandlung richtet sich nach dem betroffenen Organsystem und der Schwere der Erkrankung [3]. Eine frühe immunsuppressive Therapie stellt bislang die einzige effektive Behandlung dar [3]. Bei okulären Entzündungen sind topische Steroide indiziert, wobei die Anwendung systemischer Steroide schwerwiegenden okulären Entzündungen, einer audiovestibulären Beteiligung oder einer systemischen Vaskulitis vorbehalten sein sollte [1, 3–5]. Langfristig stellt bei Krankheitspersistenz trotz Steroidbehandlung die zusätzliche Therapie mit anderen steroidsparenden Immunsuppressiva eine sinnvolle Therapieoption dar [1, 3]. In einem weiteren Fallbericht zeigte sich, dass die Applikation von 1 % Cyclosporin A AT 4‑mal täglich über 2 Monate eine effektive Therapiemethode bei schweren Entzündungsreaktionen des vorderen Augenabschnitts assoziiert mit einem Cogan-Syndrom darstellen kann [9]. Bei dieser Patientin nahm die konjunktivale Injektion 2 Wochen nach dem Therapiestart mit 1 % Cyclosporin A AT (zusätzlich zu Atropin AT, Ofloxacin AT, Betamethason AT, Timolol AT, Dipivefrin Hydrochlorid AT sowie systemisch verabreichtem Prednisolon) ab, 5 Wochen nach Therapiestart waren die nekrotischen Areale einer nekrotisierenden Skleritis epithelialisiert, und die Schmerzsymptomatik ließ nach [9]. Zehn Wochen nach Therapiestart waren einzelne Bereiche der ausgedünnten Sklera wiederhergestellt, und der Visus hatte sich von 20/100 rechts und 20/200 links vor der Therapie auf 16/200 rechts verschlechtert und 20/20 links verbessert bei noch anhaltender vestibuloauditiver Dysfunktion [9].

In dem vorgestellten Fall zeigte sich, dass der Patient unter der Therapie mit Ciclosporin AT symptom- und beschwerdefrei wurde und in dem untersuchten Zeitraum kein Rezidiv auftrat. Zudem werden Risiken einer längerfristigen lokalen Steroidbehandlung, wie z. B. Tensioerhöhung und eine steroidinduzierte Katarakt, vermieden.

#### Prognose.

Die Krankheitsentwicklung und Prognose variieren im Einzelfall stark [5]. Bei verspäteter Diagnosestellung und Therapie kann das Cogan-Syndrom zu einem dauerhaften Hörverlust und Visuseinschränkungen führen [3, 5]. Insgesamt zeigt sich nach einer interstitiellen Keratitis jedoch selten ein dauerhafter Visusverlust [3], welcher am ehesten auf persistierenden Hornhautnarben beruht. Eine phototherapeutische Keratektomie (PTK), tiefe anteriore lamelläre Keratoplastik (DALK) oder perforierende Keratoplastik kann dann je nach Lokalisation und Ausdehnung der Narben sinnvoll sein.

## Fazit für die Praxis

- Das Cogan-Syndrom ist eine seltene Ausschlussdiagnose. Es sollte bei der Kombination von Gleichgewichtsverlust, Hörminderung und in der Regel beidseitiger interstitieller Keratitis in Betracht gezogen werden.
- Eine Therapieleitlinie besteht noch nicht. Grundsätzlich verbessert eine frühzeitige systemische Steroidtherapie in schweren Fällen die Prognose.
- Die interstitielle Keratitis sollte mit topischen Steroiden therapiert werden. Wie in dem vorgestellten Fall gezeigt, kann bei protrahiertem Verlauf eine langfristige lokale Immunsuppression mittels steroidsparender Immunsuppressiva erfolgreich sein.
